# Whole Genome Sequencing and RNA-seq-Driven Discovery of New Targets That Affect Carotenoid Synthesis in *Phaffia rhodozyma*

**DOI:** 10.3389/fmicb.2022.837894

**Published:** 2022-03-21

**Authors:** Zhihui Shi, Xiaoxian He, Hailiang Zhang, Xuena Guo, Yanfei Cheng, Xuelian Liu, Zhaoyue Wang, Xiuping He

**Affiliations:** ^1^CAS Key Laboratory of Microbial Physiological and Metabolic Engineering, State Key Laboratory of Mycology, Institute of Microbiology, Chinese Academy of Sciences, Beijing, China; ^2^College of Life Sciences, University of Chinese Academy of Sciences, Beijing, China; ^3^State Key Laboratory of Direct-Fed Microbial Engineering, Beijing DaBeiNong Science and Technology Group Co., Ltd. (DBN), Beijing, China

**Keywords:** *Phaffia rhodozyma*, carotenoids, astaxanthin, genomics, transcriptomics, *GST1*

## Abstract

Carotenoids are unsaturated compounds with terpene groups. Among them, astaxanthin has strong antioxidant properties. It is widely used in aquaculture, food, medicine, and cosmetics with a broad market prospect. *Phaffia rhodozyma* is an important microorganism that synthesizes astaxanthin, but its wild strains have low pigment content, long growth cycle, and low fermentation temperature. Therefore, it is important to research the genetic improvement of the physiological and biochemical properties of *P. rhodozyma*. In this study, the atmospheric and room temperature plasma mutagenesis technology was adopted, through the functional evolution of the carotenoid production performance; then, through the comparative analysis of the genomics and transcriptomics of the wild strain and evolved strain, the key factor *GST1* gene that affects carotenoid synthesis was discovered.

## Introduction

Carotenoids are unsaturated organic compounds with terpene groups. At present, carotenoids have been identified with more than 700 different structures, which contain multiple conjugated double bonds (Higuera-Ciapara et al., 2006). The main function of carotenoids is to protect cells from reactive oxygen damage. Among carotenoids, natural astaxanthin has a strong antioxidant ability, which can prevent cell lipid oxidation, improve organism metabolism, and have an anti-aging effect (Ambati et al., 2014; Sandmann et al., 2021). Astaxanthin plays an important role in the pigmentation of salmon and crustaceans in aquaculture. Astaxanthin can also improve the reproduction and growth performance of animals (Sandmann, 2015; Wang et al., 2021).

*Phaffia rhodozyma* (PR) was originally isolated from tree exudates in the remote areas of Japan and the west coast of North America. PR (sexual type, also named *Xanthophyllomyces dendrorhous*) synthesizes astaxanthin as the main carotenoid, which is the most promising and most economical natural source of astaxanthin. β-Carotene (a precursor of astaxanthin) is the second most abundant pigment in PR. The cells can be used directly as feed additives (Johnson and Lewis, 1979; Johnson, 2003).

In PR, the carotenoid synthesis pathway starts with the acetyl-CoA substrate, *via* the mevalonate (MVA) pathway, and by phosphorylation and decarboxylation, eight molecules of isoprene pyrophosphate (IPP, a common precursor of terpenoids) are synthesized. Two molecules of IPP are condensed to form geranyl pyrophosphate (GPP), which is then to form farnesyl pyrophosphate (FPP), geranyl geranyl pyrophosphate (GGPP), and the colorless carotenoid *cis*-phytoene. Through four-step dehydrogenation reactions and two-step cyclization reactions to synthesize β-carotene, then undergoing an oxidation reaction, astaxanthin is finally synthesized (Verdoes et al., 1999; Sieiro et al., 2003). By overcoming the limitation of a certain pathway, other reactions may become new bottlenecks because their enzymatic activity may not be able to cope with higher flow rates, which is revealed through the accumulation of intermediate products rather than final products. At present, little is known about the regulation of astaxanthin biosynthesis in PR.

Due to the commercial value of astaxanthin, many studies have focused on the mutagenesis and breeding of astaxanthin-producing strains with higher yields. Some key factors for pigment fermentation were searched, and the optimization of fermentative crafts was also adopted to increase the production of astaxanthin in PR strains. For example, oxygen and light were reported to play a synergistic effect (Breitenbach et al., 2011). Increased ventilation and low light intensity lead to a higher production of colored carotenoids and more conversion of phytoene at the expense of ergosterol (Miao et al., 2015; Gutiérrez et al., 2019). These are useful to enhance pigment synthesis; however, less crucial regulatory information based on the strain level will limit the improvement of pigment synthesis to a large extent. Hence, an investigation on the strain genetics and regulation is necessary and important. During the selection of PR strains, the colonies are orange red, and the more astaxanthin is synthesized in the cell, the darker the color. This feature makes it a high-throughput screening method for mutant strains. In this study, the carotenoid synthesis ability of PR was evolved using the atmospheric and room temperature plasma (ARTP) mutagenesis technology, and the key regulatory factors for carotenoid synthesis were searched through the whole genome sequencing (WGS) and RNA-seq of the wild strain and evolved strains. The effect of the key regulators on the physiological characteristics of PR was further investigated in order to improve the synthesis of carotenoids and guide metabolic engineering.

## Materials and Methods

### Strains and Culture Conditions

The wild-type PR strain AS2.1557 was obtained from the (China General Microbiological Culture Collection Center, Beijing, China). F94, a strain with higher carotenoid yield, was evolved and screened by ARTP mutagenesis.

The seed medium was a Yeast Extract Peptone Dextrose Medium (YPD) (composed of glucose 20.0 g, yeast extract 10.0 g, and peptone 20.0 g per liter. The fermentation medium was a YPD medium (glucose 20.0 g, yeast extract 5.0 g, and peptone 3.0 g per liter) or a Yeast Malt Agar (YM) medium (malt extract 3.0 g, yeast extract 5.0 g, and peptone 3.0 g per liter).

All experiments were conducted in a shaking flask culture in 250 ml flasks containing a fixed liquid volume of 20 ml. PR and F94 cells were, respectively, transferred from 4° YPD slants to fresh YPD and cultured at 20° for 48 h. Ten percent of the preincubation broth was inoculated to the seed medium for another 24 h, and 10% of the broth from the previous step was further inoculated to YPD for another 24 h to produce a seed culture. Ten percent of the seed culture was then inoculated to a fermentation medium, and the overall fermentation period was 84 h on a rotary shaker with 200 rpm at 20°; all experiments were performed in triplicate. The dry weight of cells was determined by centrifuging 5 ml broth at 12,000 rpm, rinsing with distilled water, and drying at 85° until it attains constant weight (∼15 h).

### Carotenoid and Astaxanthin Measurement

About 5 ml broth was centrifuged at 12,000 rpm for 1 min and washed with distilled water. Cell pellets were mixed with 1.8 ml hydrochloric acid (3 M), fully shaken and soaked for 30 min, and then incubated in a boiling water bath for 8–9 min. After observing that the cells became flocculent, they were immediately cooled down in an ice bath. The cell pellets after distilled water washing were extracted with acetone, then shaken and extracted for 15 min in the dark, until the cells were colorless. They were centrifuged at 8,000 rpm for 5 min, and the supernatant was taken.

Carotenoids were measured at a wavelength of 474 nm using a UV spectrophotometer. The calculation of carotenoid content were as described in the literature (Jiang et al., 2017).

Astaxanthin was analyzed quantitatively by HPLC on an Eclipse Plus C18 column (250 × 4.6 mm; 5 μm; Agilent, Beijing, China), with a temperature of 30°, flow rate of 1.0 ml/min, and wavelength of 478 nm. The mobile phase consisted of 90% methanol and 10% acetonitrile. Astaxanthin was identified based on the retention time in comparison with standard astaxanthin (Aladdin; Shanghai).

### Atmospheric and Room Temperature Plasma Mutagenesis

The helium-based ARTP mutation breeding system (ARTP-IIS) was from Si Qing Yuan Biotechnology Co., Ltd. (Wuxi, China). Ten microliters of approximately 2.5 × 10^8^ CFU/ml of PR strain were uniformly coated on sterile copper slides, which were then placed in the ARTP chamber for mutagenesis. The distance between the slides and the plasma emitter was adjusted to 2 mm, the output power was 120 W, and the flow rate of the carrier gas (helium) was 10.0 L/min. The mutagenic exposure time was set at 0–80 s. The treated cells were serially diluted to an appropriate concentration and inoculated onto a YPD agar to determine cell survival rates. The lethality rate was calculated as follows: lethality rate = (1 - N1/N0) × 100%, where N0 is the colony number of the control and N1 is the colony number of the mutants.

### General DNA and RNA Manipulations

The general DNA manipulations in *Escherichia coli* or *Saccharomyces cerevisiae* were partly performed according to the standard methods (Adams et al., 1997; Russell and Sambrook, 2001), and the voltage for transformation was changed to 2 kV in PR.

Total RNA extract: The cells of PR and F94 in 600 μl culture liquid were harvested by centrifugation (12,000 rpm, 1 min), frozen immediately in liquid nitrogen, and stored at −80°C. Liquid nitrogen grinding was done to break the cell wall; RNA extraction was performed using the TRIzol Reagent (Invitrogen) according to the manufacturer’s instructions. The total RNA concentration and purity were determined by using the NanoDrop instrument.

Plasmid construction: All the plasmids used in this study are described in Supplementary Table 1. Plasmid Ycp50-G, *E. coli*-yeast shuttle vector with Amp*^r^* and G418*^r^*. For expression of glutathione S-transferase (*GST1*), plasmid YCp-TA-GST1 was constructed with the *GST1* regulated by promoter *TEF1* and terminator *ADH1*. The primers used in the experiment are shown in Supplementary Table 2.

Real-time polymerase chain reaction (RT-PCR): RT-PCR analyses were performed with the LightCycler96 software SW 1.1 (Roche, Beijing, China), using RNA samples as a template. Dissociation curves were constructed to test amplification validity. Target genes were obtained from the NCBI^1^. Actin was used as a control gene. Relative gene expression was calculated by the 2^–△△^*^CT^* (cycle threshold) method using Sequence Detection software program v1.2.2 (Applied Biosystems, Beijing, China). Each RT-PCR analysis was run in quadruplicate for test consistency. qRT-PCR verification RNA-Seq result analysis is shown in Supplementary Figure 1.

### Whole Genome Resequencing

*Phaffia rhodozyma* and F94 strains were selected and their genomic DNA were sent to Nuohe Zhiyuan Biotechnology Co., Ltd. (Beijing, China) for whole genome resequencing. The SAMTOOLS bioinformatics tool was used for comparing the samples with the reference genome Xden1 GCA_001007165.2.

### Transcriptome Analysis (RNA-seq)

*Phaffia rhodozyma* and F94 were, respectively, grown in the YPD or YM medium for 42 h at 20°. The subsequent procedures for RNA sequencing were conducted by Anoroad (Beijing, China). Clean data were *de novo* assembled by the Trinity software program^2^. The genes with fold changes > 1.5-fold were functionally classified using the Munich Information Center for Protein Sequences’ FunCat. There were four parallel settings for each sample. Parallelism was demonstrated by PCA analysis (Supplementary Figure 2).

## Results and Discussion

### Atmospheric and Room Temperature Plasma Mutagenesis of *Phaffia rhodozyma* and Performance Analysis of the Mutagenized Strains

**FIGURE 1:**
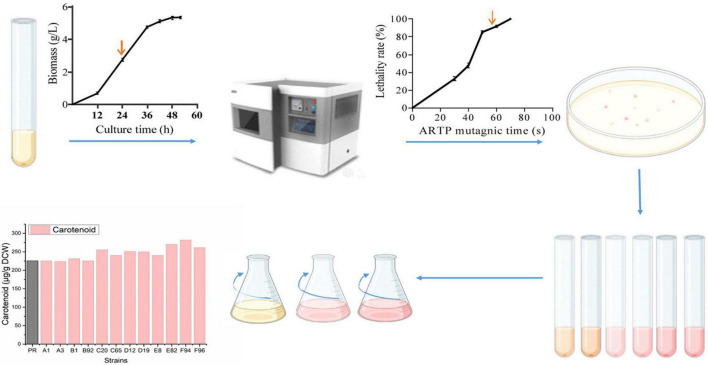
Mutagenesis flow chart. Firstly, the growth curve of wild strain PR was measured in Yeast Extract Peptone Dextrose Medium (YPD), indicating that 24 h is the logarithmic phase. Next, it was determined that the lethality rate was 85% when the ARTP mutagenesis treatment time was 50 s. Then, the strain suspension was spread on a solid medium; large and red colonies were selected. There was a preliminary screening at the test tube level, using carotenoids as indicators. Finally, rescreening was performed at the shake flask level to obtain mutagenic strains.

**FIGURE 2:**
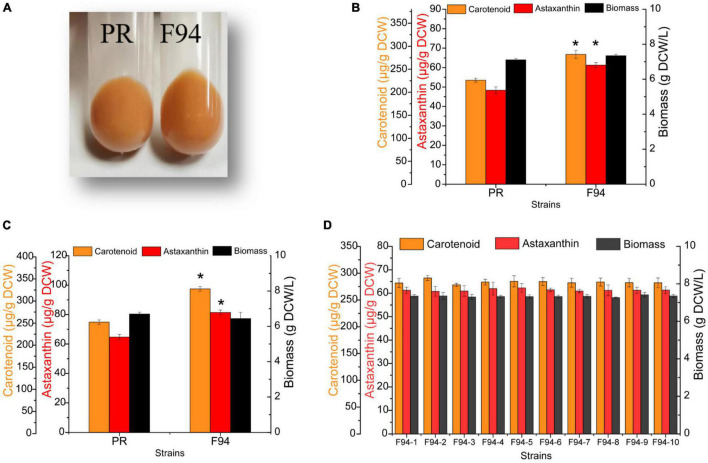
Analysis of growth performance, carotenoid synthesis ability, and stability of mutagenized strains. **(A)** Comparison of the color of PR and F94 strains. **(B)** Analysis of growth and pigment content of PR and F94 strains in YPD medium. **(C)** Analysis of growth and pigment content of PR and F94 strains in Yeast Malt Agar (YM) medium. **(D)** Stability analysis of F94 strain. Statistical analysis was performed using Student’s *t*-test (*p < 0.05).

The wild strain PR after 24 h of cultivation was collected for the ARTP treatment for about 50 s (the lethality rate was about 85%). From about 10,000 mutants, 500 colonies with darker colors were selected (Supplementary Figure 3). By a preliminary comparison of the cell growth and pigment synthesis ability of the mutants, the F94 strain was found fine (Figure 1). From the cell pellets’ color, it was shown that the evolutionary strain F94 was darker than that of the strain PR (Figure 2A). Under two culture conditions to compare the strains’ performance, it was found that after being fermented in the YPD medium for 84 h, the carotenoid content and astaxanthin content in the F94 strain, respectively, increased by 24% [225.71–281.90 μg/g dry cell weight, NAD+: Nicotinamide adenine dinucleotide (DCW)] and 27% (48.33–61.26 μg/g DCW) compared with that in the wild strain PR (Figure 2B). In the YM medium, the carotenoid content and astaxanthin content of strain F94 increased by 30% (249.49–324.82 μg/g DCW) and 26% (64.71.33–81.43 μg/g DCW), respectively (Figure 2C). The YM medium is more conducive to the synthesis of strains of carotenoids, compared with the YPD medium.

Stability was significant for the microbial strain, especially for mutated strains; hence, the F94 strain was cultivated for 15 generations before the later omics analysis. The results of the performance comparison among the multiple single colonies of the 15th generation of F94 showed that the evolutionary strain was stable no matter in cell growth or in the synthesis of carotenoid and astaxanthin (Figure 2D).

### Omics Analysis of Mutant F94 and Wild Strain *Phaffia rhodozyma*

*Phaffia rhodozyma* and F94 were selected for the whole genome resequencing, and SAMTOOLS was used for bioinformation analysis using Xden1 GCA_001007165.2 as the reference genome. The same variation information compared with the reference genome was removed from the genome information of PR and F94, and the rest was the mutation information of the F94 strain compared with the original strain PR. The resequencing analysis showed that 28 genes had single-nucleotide polymorphism (SNP) mutations in the F94 strain, 37 genes had small fragment insertion and deletion (SNP_Indel) mutations, 49 fragments had structural variations, and 222 genes had copy number variations. The specific mutation information is in the appendix (Supplementary Tables 3–6). It is limited in that there is only protein ID and no gene ID in the reference genome in the current database, so the mutated genes cannot be enriched, and large fragments of structural variation cannot be located on chromosomes.

**FIGURE 3:**
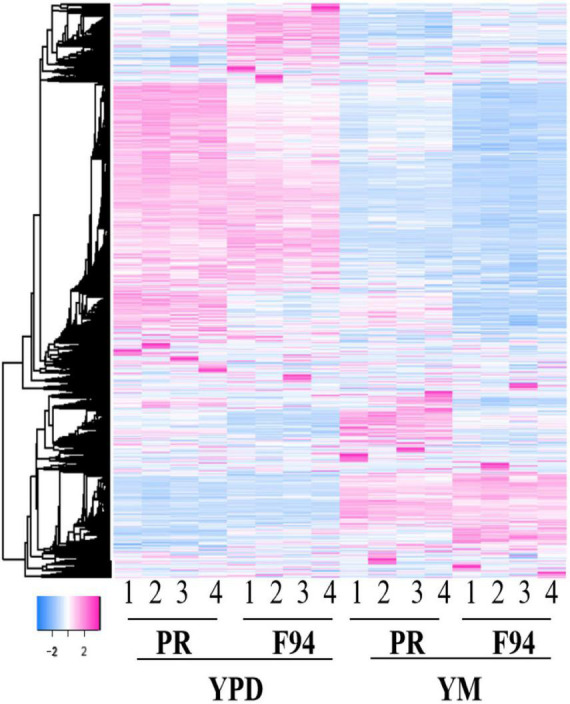
Differential gene cluster map. The heatmap of the enrichment of the differentially expressed genes of F94 compared with the wild strain PR and the strains under the Yeast Malt Agar (YM) treatment compared with the YPD. The enrichment illustrates good four repetitions. The legend of the color bar displays the log2 values of the fold change of each replicate of each gene, from –2 to 2.

We further used the RNA-Seq technique to investigate the genomic transcription changes in the mutant strain F94 compared to wild strain PR under different media. The differences of the 4 groups were given after RNA-seq analysis, including the differences of the F94 strain, respectively, under the YM and YPD media (FM_FD), among which, 819 genes were upregulated, while the remaining 485 genes were downregulated, the differences of the PR strain under the YM and YPD medium (PM_PD) with 723 of upregulated genes and 353 of downregulated genes, differences between F94 and PR under the YPD medium (FD_PD), with 153 upregulated and 137 downregulated genes, differences between F94 and PR under the YM medium (FM_PM), among which, 138 genes were upregulated and 226 genes were downregulated. R software is used for the cluster analysis of differentially expressed genes. The clustering results of the differentially expressed genes in each control group showed that the mutant strains had significant differences in transcription levels compared with wild strains, and the transcription levels of the same strain in different media were also significantly different (Figure 3). The difference coverage of the FM_PM group is broader than that of FD_PD; hence, the following analysis is mainly based on the differentially expressed genes of the F94 strain and the wild strain PR under the YM condition (FM_PM), and the data from YPD serves as a supplement. The differences in the transcription level caused by the different medium composition is mainly from the PR strain (PM_PD), and the data from F94 serve as a supplement.

### Transcriptional Profiling of F94 Compared to *Phaffia rhodozyma* Strain Fermented in Yeast Malt Agar Medium

**FIGURE 4:**
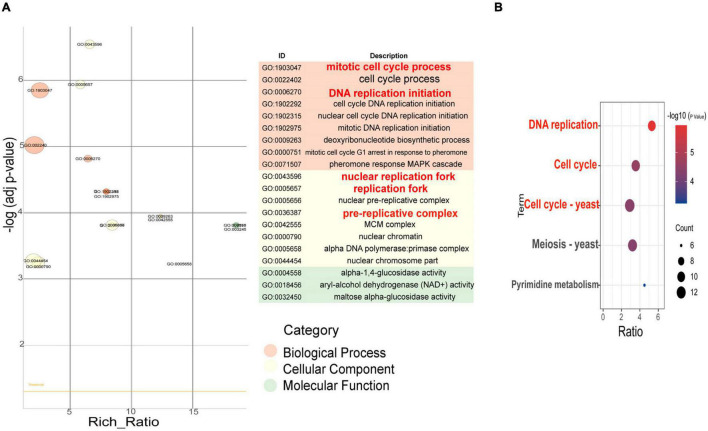
GO enrichment item and KEGG enrichment pathway *q* value scatter plot of differentially expressed genes between F94 strain and PR strain. **(A)** GO enrichment item *q* value scatter plot. **(B)** KEGG enrichment pathway *q* value scatter plot.

The transcription levels of 364 genes in the F94 strain were changed, of which 138 genes were upregulated and 226 genes were downregulated. According to the GO (Gene Ontology) enrichment analysis of differential genes, the number of differential genes in each item was analyzed by a hypergeometric test, and the significantly enriched GO items were found (with Q < 0.05 as the threshold, and the GO term satisfying this condition as the significantly enriched GO entries). In the metabolic process category, only alpha-1,4-glucosidase activity, aryl-alcohol dehydrogenase (NAD+) activity, and maltose alpha-glucosidase activity were enriched. The nuclear replication fork, replication fork, nuclear prereplication complex, prereplication complex, MiniChromosome Maintenance (MCM) complex, nuclear chromatin, alpha DNA polymerase:primase complex, and nuclear chromosome part were enriched in the cell components. In the molecular functional categories, numerous entries were shown, including mitotic cell cycle processes, cell cycle processes, DNA replication initiation, cell cycle DNA replication initiation, nuclear cell cycle DNA replication initiation, mitotic DNA replication initiation, deoxyribonucleotide biosynthesis, mitotic cell cycle G1 arrest in response to pheromone, and pheromone response MAPK cascade (Figure 4A). Taking Q < 0.05 as the standard, the enrichment analysis of each pathway in KEGG (Kyoto Encyclopedia of Genes and Genomes) was carried out by a hypergeometric test, and the significant enrichment of the pathway from differentially expressed genes was found. In the mutagenic strain F94, significant changes have taken place in the following aspects (Figure 4B): DNA replication, cell cycle, meiosis, pyrimidine metabolism, and so on. In pyrimidine metabolism, the genes encoding carbamyl phosphate synthase [EC:6.3.5.5], cytidine triphosphate (CTP) synthase [EC:6.3.4.2], ribonucleotide reductase class II [EC:1.17.4.2], and 2′-deoxyuridine 5′-triphosphate (dUTP) pyrophosphatase [EC:3.6.1.23] were downregulated. Downregulated PRPS (ribose-phosphate pyrophosphokinase) catalyzes the formation of PRPP (5-phosphate ribose-1-pyrophosphate) from ribose-5-phosphate and ATP. PRPP is a very important precursor of nucleotide synthesis. In DNA replication, there were ten genes such as *MCM2, MCM3, MCM6, MCM7, PCNA*, and *POLA1* that were downregulated. A total of 13 genes in the cell cycle such as *MCM1, MCM2, MCM3, MCM5, MCM6, MCM7, MAD3*, and *BUB1* and 12 genes in mitosis such as *SMC1, SMC3, ESP1*, and *BUB1* were downregulated. The results of GO enrichment and KEGG enrichment indicated that the transcription levels of the mutant strain were changed in multidimension not only in molecular function and metabolic processes but also in the cell cycle, mitosis, and other cellular behaviors due to the change of genomic information after ARTP mutagenesis.

### Changes in Metabolic Flux of Acetyl-CoA in Strain F94

**FIGURE 5:**
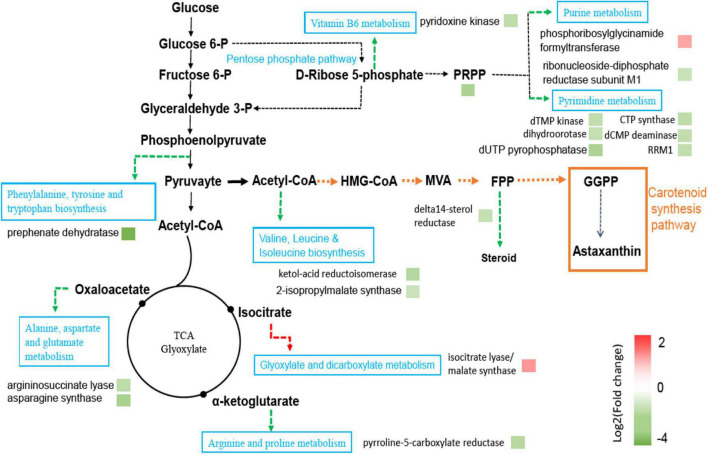
Pathways of significantly differentially expressed genes between F94 strain and PR strain. Protein names were inferred from KEGG orthologs. Upregulation of genes encoding proteins were shown in red and downregulation of genes encoding proteins were in green.

Carotenoids are an unsaturated terpene compound. Acetyl-CoA is the basic precursor for the synthesis of terpenes. Acetyl-CoA is the key molecule of central carbon metabolism and participates in a variety of metabolic processes. During the analysis of the KEGG pathway map, it was found that the metabolic flux of acetyl-CoA in the mutant strain F94 might have changed. As shown in Figure 5, the downregulation of genes encoding chorismate mutase [EC:5.4.99.5] and pre-benzoate dehydratase [EC:4.2.1.51] in the phenylalanine, tyrosine, and tryptophan biosynthesis metabolic pathway might cause more phosphoenolpyruvate to flow to pyruvate. The gene encoding pyridoxine kinase [EC:2.7.1.35] in the Vitamin B6 metabolism pathway was downregulated, which reduced carbon loss in the branching pathway and flowed more to pyruvate. Those genes encoding arginine succinate lyase [EC:4.3.2.1] and pyrroline-5-carboxylate reductase [EC:1.5.1.2] in the arginine and proline metabolism pathway and the genes encoding asparagine synthase and argininosuccinate lyase [EC:4.3.2.1] in the alanine, aspartate, and glutamate metabolism pathway were also downregulated, which might reduce the flow of acetyl-CoA to tricarboxylic acid cycle. However, the genes encoding isocitrate lyase [EC 4.1.3.1] and malate synthase [EC:2.3.3.9] in glyoxylic acid and glyoxylate and dicarboxylate metabolism were upregulated. It has reduced the carbon loss of acetyl-CoA and increased the production of α-sandalene in yeast by knocking out the two genes of the glyoxylic acid cycle, citrate synthase, and malate synthase (Chen et al., 2013). The downregulation of the genes encoding ketol-acid reductoisomerase [EC:1.1.1.86] and 2-isopropylmalate synthase [EC:2.3.3.13] in the leucine and isoleucine biosynthesis pathway might cause more acetyl-CoA to flow to the MVA pathway. The gene encoding Delta14-sterol reductase [EC:1.3.1.70] in steroid biosynthesis pathway was downregulated. The steroid biosynthesis pathway competes with the carotenoid biosynthesis precursor FPP. The decrease of steroid biosynthesis might cause more FPP to flow to the carotenoid biosynthesis pathway.

In addition, it was found that the gene encoding the ABC transporter was upregulated in this study. The ABC transporter played an important role in the accumulation of secondary metabolites and transmembrane transport. Some researchers found that the addition of gibberellin induced the excessive synthesis of astaxanthin. By combining the analysis of transcriptome and metabolome, they also reported that the gene encoding the ABC transporter was upregulated and the proposed strategy of transporter engineering to increase the production of astaxanthin (Liu et al., 2021).

### Transcriptional Profiling of *Phaffia rhodozyma* Strain Fermented in Yeast Malt Agar Compared to That in Yeast Extract Peptone Dextrose Medium

The fermentation in different media showed that the YM medium was more conducive to the synthesis of carotenoids by PR. Besides the analysis of different strains, RNA-seq was also adopted to analyze the transcriptional changes of wild strain PR in the YM medium and YPD medium. The transcription level of 1,076 genes changed, of which 723 genes were upregulated and 353 genes were downregulated in YM.

**FIGURE 6:**
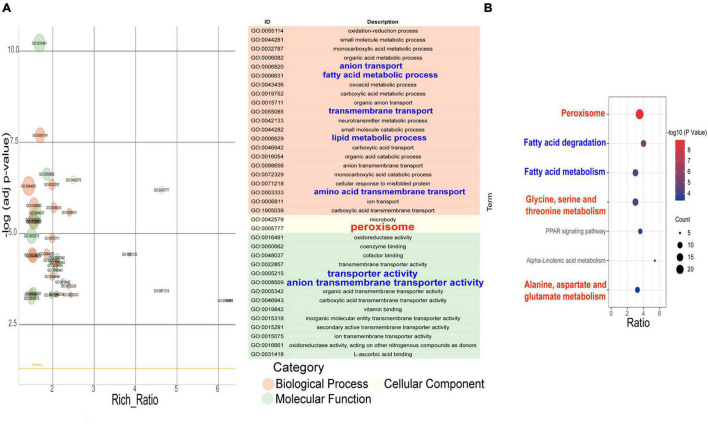
GO enrichment item and KEGG enrichment pathway *q* value scatter plot of differentially expressed genes that the strains under the YM medium compares with the YPD medium. **(A)** GO enrichment item *q* value scatter plot. **(B)** KEGG enrichment pathway *q* value scatter plot.

The results of the GO enrichment of the differential genes of the PR strain under the conditions of the YM medium compared to the YPD medium showed that the metabolic processes were enriched into the oxidation–reduction process, fatty acid metabolic process, oxoacid metabolic process, transmembrane transport, lipid metabolic process, protein refolding, amino acid transmembrane transport, etc. Peroxisome was enriched in the cell components. The molecular functional categories were enriched in oxidoreductase activity, coenzyme binding, cofactor binding, transmembrane transporter activity, vitamin binding, the hydrolase activity on glycosyl bonds, L-ascorbic acid binding, and so on (Figure 6A). GO enrichment also showed that different nutrients in the culture medium led to the changes in intracellular metabolic activity, peroxisome, transporter activity, and coenzyme/cofactor binding.

The results of the KEGG pathway enrichment of the differential gene in the PR strain under different media conditions (Figure 6B) showed that the PR cells in the YM medium had undergone significant changes in the following aspects: peroxisome, fatty acid metabolism, glycine/serine and threonine metabolism, the PPAR signal pathway, α-linolenic acid metabolism, alanine/aspartic acid and glutamate metabolism, arginine biosynthesis, and so on.

Peroxisomes are indispensable organelles in cells, which play a key role in redox signal transduction and lipid homeostasis. They contribute to many important metabolic processes, such as fatty acid oxidation, lipid biosynthesis, and free radical detoxification. Under the YM condition, 20 genes such as *PEX5, PEX3, PEX14, PEX16*, and *PXMP4* were upregulated and 3 genes, *AGXT, HMGL*, and *EPHX2*, were downregulated in the peroxisome metabolic pathway. A 3-hydroxy-3-methylglutaryl-CoA lyase (HMGL) catalyzes the formation of ketones from 3-hydroxy-3-methylglutaryl-CoA (HMG-CoA) in organisms. HMG-CoA is an important intermediate in the MVA pathway. The downregulation of HMGL makes more HMG-CoA flow to the MVA pathway, resulting in the synthesis of more carotenoids. The genes in the MVA pathway were overexpressed and located in the peroxisome of *Yarrowia lipolytica*. The engineering strain MP2 could produce MVA using fatty acids, and the yield was twice as high as that using the glucose in the cytoplasm (Jiang, 2019). It has not been reported that the increase of carotenoid synthesis is through the upregulation of the genes in peroxisome. Based on our transcriptome analysis, it was speculated that a strategy using peroxisome engineering might increase carotenoid synthesis in PR.

Studies have shown that fatty acid saturation affects the synthesis of astaxanthin (Liu et al., 2021), and fatty acid metabolism pathways are related to the synthesis of carotenoids. In our study, nine genes encoding long-chain acyl-CoA synthetase [EC:6.2.1.3], acyl-CoA oxidase [EC:1.3.3.6], acetyl-CoA acyltransferase [EC:2.3.1.16], and acyl-CoA dehydrogenase [EC:1.3.99] were upregulated in the fatty acid degradation pathway. Two genes encoding alcohol dehydrogenase 1 [EC:1.1.1.1] and aldehyde dehydrogenase (NAD+) [EC:1.2.1.3] were downregulated. The enhancement of the fatty acid degradation pathway should contribute to the synthesis of carotenoids.

In glycine, serine, and threonine metabolism, eight genes were upregulated including genes encoding 3-bisphosphoglycerate-independent phosphoglycerate mutase [EC:5.4.2.12], L-serine/L-threonine ammonia lyase [EC:4.3.1.17, 4.3.1.19], and primary-amine oxidase [EC:1.4.3.21]. *AGXT* for alanine-glyoxylate transaminase[EC:2.6.1.44]/serine-glyoxylate transaminase [EC:2.6.1.45]/serine-pyruvate transaminase [EC:2.6.1.51], gene for aminomethyltransferase [EC:2.1.2.10], and five other genes were downregulated. In alanine, aspartate, and glutamate metabolism, eight genes were downregulated such as the genes for argininosuccinate synthase [EC:6.3.4.5], alanine-glyoxylate transaminase/(R)-3-amino-2-methylpropionate-pyruvate transaminase [EC:2.6.1.44 2.6.1.40], argininosuccinate lyase [EC:4.3.2.1], and succinate-semialdehyde dehydrogenase/glutarate-semialdehyde dehydrogenase [EC:1. 2.1.16 1.2.1.79 1.2.1.20].

Coincidentally, in the analysis of the transcription level of the mutant strain F94, the transcription level of argininosuccinate lyase was also downregulated. Amino acid metabolism is closely related to acetyl-CoA. Since acetyl-CoA is the precursor of the MVA pathway, we speculate that the carotenoid synthesis in PR may improve by changing the amino acid pathway, for example, glycine, serine, and threonine metabolism; alanine, aspartate, and glutamate metabolism; and arginine and proline metabolism, as mentioned above.

### The Effect of *GST1* Gene on the Growth Performance and Carotenoid Synthesis of *Phaffia rhodozyma*

Based on the intersection clustering of the four groups of differential genes in transcriptome analysis, 22 common differentially expressed genes were obtained from the Venn plot result (Supplementary Figure 4). Then, after combining the genomic SNP variation information (Supplementary Table 7), it was found that the gene *GST1* encoding glutathione S-transferase [EC: 2.5.1.18] had SNP mutations at four sites, and its expression level presented enhancement. In addition, the transcription level of *GST*1 in the YM medium is also upregulated, indicating that the difference between the mediums can be reversed to influence carotenoid synthesis. Comprehensively considering the probable effect of the above factors on the physiological and biochemical properties of PR, the *GST1* gene was chosen for further investigation.

In genome resequencing analysis, four mutations were found in *GST1* (A0317) in mutant strain F94, one of which was synonymous mutation and the other three sites were missense mutations, which were the 164 site of Pro mutating to Leu, 171 site of Thr to Lys, and 194 site of Ala to Val. In transcriptome analysis, the *GST1* level of strain F94 was upregulated by 2.42 times compared with that of the PR strain. KEGG metabolic pathway analysis showed that glutathione S-transferase was involved in the metabolism of xenobiotics by cytochrome P450. In eukaryotes, glutathione S-transferase is a superfamily, which is a multifunctional enzyme that participates in detoxification by catalyzing the binding of electrophilic compounds with glutathione (Jakoby, 1978). In terms of the taxonomic structure, the classification of glutathione S-transferase from bacteria, mammals, and higher plants is relatively clear. However, due to the diversity of fungal glutathione S-transferase, it is difficult to classify, and there is less research in fungus. Gtt1p, Gtt2p, Gto1p, Gto2p, and Gto3p are currently found in the model fungus *Saccharomyces cerevisiae*, which belongs to the glutathione S-transferase family. The research on glutathione S-transferase in PR is almost blank (Choi et al., 1998; Ma et al., 2009) till now.

**FIGURE 7:**
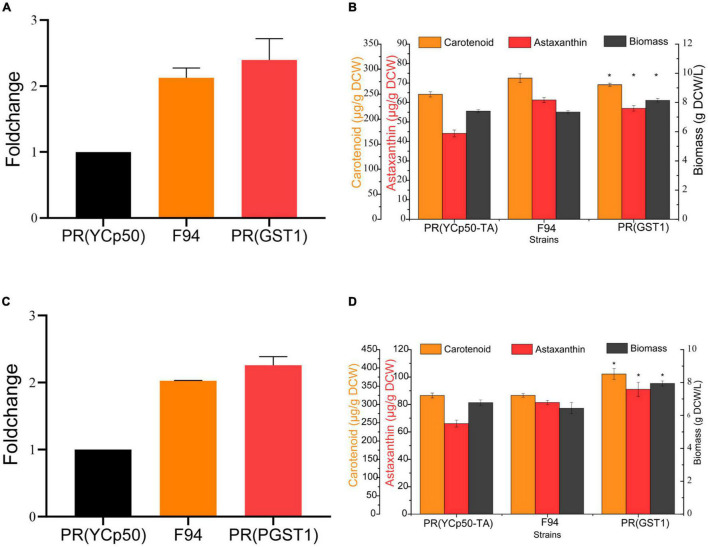
Analysis of *GST1* transcription level and the growth and carotenoid synthesis ability of *GST1* overexpression strain. **(A)** Analysis of GST1 transcription level of PR (YCp-TA), F94, and PR (GST1) strains in YPD media. **(B)** Analysis of the growth and carotenoid synthesis ability of PR (YCp-TA), F94, and PR (GST1) strains in YPD media. **(C)** Analysis of *GST1* transcription level of PR (YCp-TA), F94, and PR (GST1) strains in YM media. **(D)** Analysis of the growth and carotenoid synthesis ability of PR (YCp-TA), F94, and PR (GST1) strains in YM media. Statistical analysis was performed using Student’s *t*-test (*p < 0.05).

In order to investigate the effect of *GST1* on PR performance, using the complementary DNA (cDNA) of the PR strain as a template, the *GST1* gene with no intron was cloned, and its overexpression strain PR (GST1) was further constructed. The results of qRT-PCR showed that the level of *GST1* transcription in F94, PR (GST1) strains was twice as high as that in the control strain PR (YCp-TA) with an empty vector (Figures 7A,C), and there was no difference in the *GST1* transcription level between F94 and PR (GST1) strains by the *T*-test. The fermentation results of these three strains in YPD and YM media showed that when fermented in the YPD medium for 84 h, the biomass of PR (GST1) increased by 10%, the carotenoid content increased by 17%, and the astaxanthin content increased by 29% than that of PR (YCp-TA) (Figure 7B). When fermented in the YM medium for 84 h, the biomass of PR (GST1) increased by 17%, compared to the control strain PR (YCp-TA), the content of carotenoids increased by 18%, and the astaxanthin content increased by 40% than that of the control strain (Figure 7D), indicating that *GST1* overexpression not only promoted the growth of PR but also enhanced the synthesis of carotenoid and astaxanthin. The results also showed that the overexpression of *GST1* in the wild strain could promote the strain to synthesize pigment, reaching to the level as the mutant strain F94, which indicates that glutathione S-transferase plays an important role in the synthesis of astaxanthin by PR.

The glutathione S-transferase family exists in almost all plants. There are as many as 90 genes encoding glutathione S-transferase in plants. Most genes are differentially expressed under stress induction, and they play a role in the enzymatic removal of reactive oxygen. Under salt, low temperature, drought, heavy metal stress, and other conditions, glutathione S-transferase can remove active oxygen and protect the plant cell membrane structure and protein activity. The ThGSTZ1 gene of the bristly willow was ever overexpressed in *Arabidopsis thaliana*; it was found that under the salt stress condition, the transgenic plants had lower water loss rate, increased biomass, and significantly lower hydrogen peroxide and superoxide anion levels in the body than that of the wild type (Yang et al., 2014). In our study, the overexpression of glutathione S-transferase in the PR strain led to the increased biomass, carotenoid, and astaxanthin content. The increased growth was probably owing to the fact that glutathione S-transferase could eliminate those harmful substances produced by the metabolism of the cells in the later fermentation period; hence, the cells were not destroyed by toxic substances. Since astaxanthin is also an antioxidant, the glutathione S-transferase overexpression strain might mainly rely on glutathione to remove active oxygen; as a result, the astaxanthin consumption in the cells were reduced and its content increased.

## Conclusion

In summary, a new and significant target *GST1* gene was found through the genome resequencing and transcriptome sequencing analyses of the wild strain PR and evolutionary strain F94 obtained by ARTP mutagenesis. We have verified that *GST1* plays an important role in the synthesis of astaxanthin and growth regulation of *P. rhodozyma*. Its influence mechanism needs to be further studied. Based on the analysis of differentially expressed genes derived from the differential pigment synthesis by the *P. rhodozyma* in different media, it was discovered that the metabolic pathways in peroxisomes and synthesis pathways of amino acids may be related to the synthesis of carotenoids in *P. rhodozyma*. The discovery of the above crucial target gene and pathways is helpful to understand the pigment synthesis in *P. rhodozyma*. It suggests that further evolution and mining the key information of the genome and transcriptome are of great significance for an in-depth elaboration of the pigment synthesis and molecular regulation mechanism, which can hopefully guide the efficient synthesis of carotenoids in *P. rhodozyma*.

## Data Availability Statement

The datasets presented in this study can be found in online repositories. The names of the repository/repositories and accession number(s) can be found in the article/Supplementary Material.

## Author Contributions

ZS: designing experiments and implementation, data curation, writing original draft, review, and editing. XXH: data curation. HZ and XG: ARTP mutants screening assistance. YC and XL: conception and methodology. ZW: conception, supervision, writing, review, and editing. XPH: conception, supervision, and funding acquisition. All authors agreed to be accountable for the content of the work.

## Conflict of Interest

HZ and XL were employed by Beijing DaBeiNong Science and Technology Group Co., Ltd. (DBN). The remaining authors declare that the research was conducted in the absence of any commercial or financial relationships that could be construed as a potential conflict of interest.

## Publisher’s Note

All claims expressed in this article are solely those of the authors and do not necessarily represent those of their affiliated organizations, or those of the publisher, the editors and the reviewers. Any product that may be evaluated in this article, or claim that may be made by its manufacturer, is not guaranteed or endorsed by the publisher.

## Footnotes

1
ncbi.nlm.nih.gov/

2
github.com/trinityrnaseq/trinityrnaseq/wiki
