# Supplementary material for: Whole Genome Sequencing and RNA-seq-Driven Discovery of New Targets That Affect Carotenoid Synthesis in Phaffia rhodozyma
Source: Front Microbiol. 2022 Mar 21;13:837894. doi: 10.3389/fmicb.2022.837894 (PMC8978057; doi:10.3389/fmicb.2022.837894)
Supplement: Supplementary file 1 [file Data_Sheet_1.PDF]

1 Supplementary Tables

Table S1 Strains and plasmids used in this work

Strain/Plasmid	Description	Reference
Strains		
E. coli DH5α	*sup*E44 Δ*lac*U169(*φ*80*lac*ZΔM15) *hsd*R17 *rec*Al *end*Al *gyr*A96 *thi*-1 *rel*Al	Stratagene
Phaffia rhodozyma AS2.1557	Wild strain	CGMCC
S.cerevisiae YS58	MATα flo1 leu2-3,112 his4-519 trp1-719 ura3-52	Teunissen et al.，1993
F94	Mutagenic strains	This study
PR（YCp-TA）	PR with vector YCp-TA	This study
PR（GST1）	PR with vector YCp-TA-GST1	This study
Plasmids		
YCp50-G	E.coli-Yeast shuttle vector, Amp^r and $G418^r$	This laboratory
YCp-TA	YCp50-G derivative with TEF1 promoter and ADH1 terminator, Amp^r and $G418^r$	This study
YCp-TA-GST1	YCp50-G derivative with insertion of 1.5 kb *GST1* expression cassette with *TEF1P* and *ADH1T*, Amp^r and $G418^r$.	This study

Table S2 Primers used in this study

Primer	Sequences（5'-3'）	Purpose
GST1-F	CAATCTAATCTAAGTTTTAATTACAAATCTA GAATGAGCACCGAACTGAAGAAAC	PCR of *GST1*
GST1-F	TAATAATAAAAATCATAAATCATAAGAAATT CGCGGATCCTCACTTCGTGCTCGGAGG	
TEF1P-F	GTATCACGAGGCCCTTTCGTCTTCAAGAATT CGAATCCTTACATCACACCCAAT	fusion PCR of *TEF1*P
TEF1P-R	CGGATCCAAGTGGGTAAGCAAGTTTCTAGAT TTGTAATTAAAACTTAGATTAGAT	
ADH1T-F	CAAATCTAGAAACTTGCTTACCCACTTGGAT CCGCGAATTTCTTATGATTTATGAT	fusion PCR of *ADH1*T
ADH1T-R	GGTTGAAGGCTCTCAAGGGCATCGGTCGACC CGGTAGAGATGTGG	
ACTINQPF-1	CCCCAAGGCTAACAGAGAGA	qRT-PCR

ACTINQPR-1	CAGAGGCGTACAAAGAAAGCA	
GST1QPF-2	GGGTGTTTTGGTCACCGTTC	
GST1QPR-2	CTCATCGTCGGCTCTCTCTC	
comEBQP-F	TGCATGAAGAGACGAGTGGG	
comEBQP-R	CTCGCTCTTCGCCTTTGTTG	
dUTQP-F	TCGTCCTGCTCTTCAACCAC	
dUTQP-R	GTCTCTTCCAGGCTCTCGAC	
ERG4QP-F	GAGGCAGGCAAGAAGAAGGA	
ERG4QP-R	GGGGAATCCGACCATCATCC	
STRAPQP-F	AGCGAGAATGCTACAAGGGG	
STRAPQP-R	GAGGCCGTAAGATCGACCAG	
ABCtQP-F	GCTGTCGATGAGGAGAAGGG	
ABCtQP-R	TGAGCACGGTAGTCGTCTTG	
BGLAQP-F	TCCTCGAAGATGGAGTGGATG	qRT-PCR
BGLAQP-R	TCAACGTAAACGCACCCAAA	
fucPQP-F	TCTTCTCGTCTGCGGTGTTT	
fucPQP-R	CGACGACGTAGATGAAGGCA	
LeuAQP-F	TCGTGTTCACCGCATTCTCT	
LeuAQP-R	CCCATCCTGGCTCCTTGTTT	
PrsAQP-F	CCGTGATTCCTCACTTCCCT	
PrsAQP-R	ACATGGTCACAACCAGCCTT	
PyrGQP-F	GTTTGGGAGAGGCACAGACA	
PyrGQP-R	TCAACTGCTCTCCGTCCTTG	
SF3B1QP-F	TGGGATCAAACACCGGTCAA	
SF3B1QP-R	TCGGCCATCATACCAAAGGG	
STKCQP-F	GCATCGCATCAGGACAACAC	
STKCQP-R	GTTGAGCTCTTGCGTCGTTC	

Table S3 Summary table of SNP mutation information of F94 strain

Chr_id	Gene_id	Protein
Scaffold_162	A0090	40s ribosomal protein s14
Scaffold_162	A0183	Diacylglycerol o-acyltransferase
Scaffold_162	A0184	Conserved WD40 repeat-containing protein
Scaffold_162	A0192	Histone-like protein

Scaffold_162	A0193	Histone H4.5
Scaffold_162	A0194	Synaptobrevin-like protein
Scaffold_162	A0201	Ribosomal protein L9/RNase H1, N-terminal
Scaffold_162	A0204	Histone partial
Scaffold_162	A0205	Histone-like protein
Scaffold_162	A0206	Putative translation initiation inhibitor UK114/IBM1
Scaffold_162	A0207	Hypothetical protein
Scaffold_162	A0213	d-3-phosphoglycerate dehydrogenase 2
Scaffold_162	A0215	Possible oxidoreductase
Scaffold_162	A0223	Aquaporin (major intrinsic protein family）
Scaffold_162	A0224	Uncharacterized conserved protein
Scaffold_262	A1391	60s ribosomal protein l18
Scaffold_54	A2680	Armadillo-like helical
Scaffold_69	A2939	Mitochondrial atp epsilon chain
Scaffold_77	A3414	TPMT family
Scaffold_78	A3708	40s ribosomal protein s25
Scaffold_79	A3895	Predicted histone tail methylase containing SET domain
Scaffold_185	A0317	Glutathione S-transferase
Scaffold_22	A0430	Cytochrome c oxidase subunit 1
Scaffold_249	A0847	FOG:Transposon-encoded proteins with TYA,reverse transcriptase, integrase domains in various combinations
Scaffold_251	A1189	Retrotransposon ty1-copia subclass
Scaffold_56	A2827	Mfs monosaccharide transporter
Scaffold_56	A2828	Sugar (and other） transporter<79% Ceratobasidium sp. AG-Ba isolate JN chromosome 2>

Table S4 F94 strain Indel mutation location information table

Chr_id	Position	Type	Base	Pos_start	Pos_end	Gene_id	Protein
Scaffold_162	498953	D14	CCATCAGAATACAA	498774	498956	A0095	Uncharacterized conserved protein
Scaffold_162	498228	I3	AAG	498204	498332	A0095	Uncharacterized conserved protein
Scaffold_162	875548	D6	GCGACG	875300	875637	A0187	Opt oligopeptid transporter
Scaffold_	875745	I3	TTC	875719	875783	A0187	Opt oligopeptide transporter

162							
Scaffold_ 162	962904	D3	ACG	962691	963218	A0209	Ankyrin repeat
Scaffold_ 162	962951	D6	TAGCAG	962691	963218	A0209	Ankyrin repeat
Scaffold_ 162	1038139	I3	CAG	1037693	1038625	A0225	Hypothetical protein
Scaffold_ 189	284542	I3	TGA	284529	285116	A0390	DNA replication checkpoint mediator,MRC1domain
Scaffold_ 189	307357	I1	C	307312	307372	A0395	Tetracycline resistance protein TetB/drug
Scaffold_ 189	347917	I12	ACAGCAGCAG CA	347705	348797	A0405	Transcriptional activator FOSB/c-Fos and related bZIP transcription factors
Scaffold_ 249	1087841	I1	A	1087834	1087879	A0742	Glycoside hydrolase family 15 protein
Scaffold_ 249	1877471	I1	T	1877469	1877599	A0936	Steroid reductase required for elongation of the very long chain fatty acids
Scaffold_ 249	283928	D3	TTT	283589	283964	A1034	Glycoside hydrolase,superfamily
Scaffold_ 24	146726	I1	T	146623	146810	A1121	Nedd8 activating enzyme
Scaffold_ 262	1257510	D3	ATA	1257319	1257883	A1417	Ser thr protein phosphatase
Scaffold_ 52	1570291	I18	CTCCTCCACCA CCTCCTC	1570181	1570403	A2343	RRM motif-containing protein
Scaffold_ 52	1799949	D1	G	1799921	1800029	A2397	Uncharacterized conserved protein
Scaffold_ 52	1844966	D1	T	1844939	1845154	A2405	Alpha/beta hydrolase fold-3
Scaffold_ 52	385578	I6	TGAATC	385464	385668	A2607	Pre-mrna-splicing factor clf1
Scaffold_ 77	54154	I3	TCC	53022	54224	A3417	Synaptic vesicle protein EHS-1 and related EH domain proteins
Scaffold_ 78	1152266	I1	C	1152060	1152862	A3595	cAMP-dependent protein kinase typesI and II,regulatory subunit
Scaffold_ 78	1192377	I6	CGCCTC	1192332	1192547	A3605	Protein kinase C substrate,80KD protein,heavy chain
Scaffold_ 79	1342808	D3	AAG	1342496	1342868	A4059	Cog complex component
Scaffold_ 79	1623628	D1	A	1623624	1624488	A4129	Vacuolar sorting protein VPS45
Scaffold_ 162	724972	D30	GCACCGTAGCT GGGGCTGGCA	724217	725077	A0150	beta-1,6-N-acetylglucosaminyltransferase, contains WSC domain

Scaffold_249	1874181	I15	GAAGCGCTGTCCTCCACCTCTTCC	1873883	1874895	A0935	Uncharacterized conserved protein
Scaffold_262	242057	D15	ACTACTACTGCTGCT	241810	242324	A1548	Nucleolar GTPase/ATPase p130
Scaffold_262	243593	D9	CCTCCTCCT	243362	243961	A1549	64 kda mitochondrial nadh dehydrogenase
Scaffold_33	947694	D15	ACAACAGCAGCAACA	947440	948002	A1694	TUP1P

Chr_id	Position	Type	Base	Pos_start	Pos_end	Gene_id	Protein
Scaffold_33	1722807	I1	T	1722805	1722910	A1881	Proline oxidase
Scaffold_33	451886	I3	GGC	451097	452844	A2025	Hypothetical protein
Scaffold_52	2381763	I3	CTT	2381313	2381784	A2525	GTPase Rab2, small G protein superfamily
Scaffold_69	764352	I2	CT	764282	764366	A2927	Kinase-like protein
Scaffold_69	780136	I1	A	780120	780248	A2932	Predicted mechanosensitive on channel
Scaffold_69	1416004	D3	CTG	1415545	1416425	A3091	Protein of unknown function DUF4448
Scaffold_78	699169	D3	TCA	699151	699251	A3478	Sirtuin5 and related class III sirtuins (SIR2 family）
Scaffold_78	1356450	I4	TGTC	1356404	1356559	A3656	Hypothetical protein
Scaffold_79	1402862	D9	GAGGAGGAG	1402770	1403207	A4073	WD40 repeat protein

Table S5 F94 strain SV variant information site table

Chr1	Pos1	Orientation1	Chr2	Pos2	Orientation2	Type	Size	Score	num_Reads
Scaffold_79	761153	134+68-	Scaffold_33	375119	1+28-	CTX	-363	54	28
Scaffold_79	748719	132+377-	Scaffold_33	936050	109+279-	CTX	-363	42	21
Scaffold_33	432592	0+21-	Scaffold_78	303448	0+23-	CTX	-363	62	21
Scaffold_33	1532722	26+39-	Scaffold_262	656275	105+63-	CTX	-363	94	22
Scaffold_242	652167	631+373-	Scaffold_24	524268	1229+1015-	CTX	-363	99	401
Scaffold_54	692446	0+35-	Scaffold_124	267634	44+1-	CTX	-363	67	22
Scaffold_249	8458	0+27-	Scaffold_64	4806	85+26-	CTX	-363	75	26
Scaffold_78	1745121	24+2-	Scaffold_185	3437	49+0-	CTX	-363	42	21
Scaffold_162	142455	29+1-	Scaffold_125	44486	30+0-	CTX	-363	91	29

Scaffold_54	326399	34+1-	Scaffold_97	1	16+73-	CTX	-363	59	33
Scaffold_79	745333	2+29-	Scaffold_149	1637	1+32-	CTX	-363	99	27
Scaffold_262	654734	108+111-	Scaffold_96	1	44+74-	CTX	-363	78	20
Scaffold_33	1550783	38+16-	Scaffold_96	1	44+74-	CTX	-363	45	20
Scaffold_69	2089673	25+34-	Scaffold_46	22	51+56-	CTX	-363	59	34
Scaffold_249	1498890	27+83-	Scaffold_133	1	29+103-	CTX	-363	34	21
Scaffold_52	753734	23+0-	Scaffold_52	754158	1+26-	DEL	465	99	23
Scaffold_33	1536776	88+3-	Scaffold_33	1537276	2+40-	DEL	534	99	30
Scaffold_262	1373916	25+1-	Scaffold_262	1374224	1+25-	DEL	392	99	25
Scaffold_54	671238	23+0-	Scaffold_54	671598	1+25-	DEL	429	99	22
Scaffold_242	107791	36+2-	Scaffold_242	112874	25+24-	DEL	5238	99	23
Scaffold_79	678692	62+40-	Scaffold_79	687343	25+0-	ITX	7813	99	25
Scaffold_78	1043893	0+20-	Scaffold_78	1049761	20+8-	ITX	5074	99	20
Scaffold_262	67485	33+29-	Scaffold_262	67591	33+29-	ITX	-260	99	23
Scaffold_249	1517929	63+73-	Scaffold_33	1553194	35+46-	CTX	-373	71	27
Scaffold_79	761141	143+79-	Scaffold_262	654601	85+119-	CTX	-373	32	27
Scaffold_69	2101408	115+73-	Scaffold_162	323692	12+50-	CTX	-373	46	30
Scaffold_78	298062	34+33-	Scaffold_162	321869	89+132-	CTX	-373	77	29
Scaffold_249	2255558	759+924-	Scaffold_24	524270	1241+986-	CTX	-373	99	157
Scaffold_52	1096089	16+38-	Scaffold_124	32864	2+29-	CTX	-373	90	25
Scaffold_262	1692703	26+42-	Scaffold_77	117477	36+0-	CTX	-373	57	35
Scaffold_72	1174	1841+20-	Scaffold_148	1	1910+2596-	CTX	-373	99	1790
Scaffold_36	1421	2020+2722-	Scaffold_130	1	1094+1649-	CTX	-373	59	81
Scaffold_232	4630	1234+732-	Scaffold_188	2135	43+87-	CTX	-373	99	74
Scaffold_33	936365	116+215-	Scaffold_35	1	933+1290-	CTX	-373	99	69
Scaffold_33	938560	232+18-	Scaffold_35	1	933+1290-	CTX	-373	99	187
Scaffold_77	101	186+171-	Scaffold_35	1	933+1290-	CTX	-373	99	154
Scaffold_35	256	933+1290-	Scaffold_253	1	685+884-	CTX	-373	99	317
Scaffold_10	553	980+1222-	Scaffold_230	1	901+1397-	CTX	-373	70	38
Scaffold_199	1388	1423+1954-	Scaffold_25	55	2215+882-	CTX	-373	99	640
Scaffold_28	1596	1354+1467-	Scaffold_25	55	2215+882-	CTX	-373	99	921
Scaffold_62	74	7+394-	Scaffold_25	55	2215+882-	CTX	-373	99	388
Scaffold_62	1305	1114+222-	Scaffold_25	55	2215+882-	CTX	-373	99	182
Scaffold_232	4630	1234+732-	Scaffold_4	12	404+177-	CTX	-373	99	150
Scaffold_232	4630	1234+732-	Scaffold_70	1	40+53-	CTX	-373	99	35
Scaffold_249	433524	21+1-	Scaffold_249	433884	0+21-	DEL	433	99	20
Scaffold_249	621637	20+1-	Scaffold_249	621999	0+21-	DEL	428	99	20

Scaffold_249	1823002	24+2-	Scaffold_249	1824517	2+45-	DEL	1653	99	23
Scaffold_242	317706	40+0-	Scaffold_242	318410	0+43-	DEL	866	99	22
Scaffold_52	2135845	83+63-	Scaffold_52	2170189	31+3-	INV	9687	99	68

Table S6 F94 strain CNV mutation information table

ID	Type	Anno
A0067	duplication	{SPAC3H5.06c; KOG0970 DNA polymerase alpha, catalytic subunit L Replication, recombination and repair ;}
A0068	duplication	{SPAC29E6.08; KOG3302 TATA-box binding protein (TBP）, component of TFIID and TFIIIB K Transcription ;}
A0089	duplication	{YBR121c; KOG2298 Glycyl-tRNA synthetase and related class II tRNA synthetase J translation, ribosomal structure and biogenesis ;}
A0100	duplication	{YFL030w; KOG2862 Alanine-glyoxylate aminotransferase AGT1 R General function prediction only ;}
A0122	duplication	{YML080w; KOG2335 tRNA-dihydrouridine synthase J Translation, ribosomal structure and biogenesis ;}
A0144	duplication	{Hs4506017; KOG0371 Serine/threonine protein phosphatase 2A, catalytic subunit T Signal transduction mechanisms ;}
A0155	duplication	{Hs8923754; KOG0598 Ribosomal protein S6 kinase and related proteins RT General function prediction only ; Signal transduction mechanisms ;}
A0199	duplication	{YDR105c; KOG2592 Tumor differentially expressed (TDE） protein S Function unknown ;}
A0232	duplication	{7293606; KOG3344 40s ribosomal protein s10 J Translation, ribosomal structure and biogenesis ;}
A0234	duplication	{SPBC1711.10c; KOG2834 Nuclear pore complex, rNpl4 component (sc Npl4） YU Nuclear structure ; Intracellular trafficking, secretion, and vesicular transport ;}
A0278	duplication	{Hs4557719; KOG0967 ATP-dependent DNA ligase I L Replication, recombination and repair ;}
A0154	deletion	{SPBC215.05; KOG2711 Glycerol-3-phosphate dehydrogenase/dihydroxyacetone 3-phosphate reductase C Energy production and conversion ;}
A0183	deletion	{Hs14211871; KOG0831 Acyl-CoA:diacylglycerol acyltransferase (DGAT） I Lipid transport and metabolism ;}
A0187	deletion	{YJL079c; KOG3017 Defense-related protein containing SCP domain S Function unknown ;}
A0194	deletion	{SPAC6G9.11; KOG0860 Synaptobrevin/VAMP-like protein U Intracellular trafficking, secretion, and vesicular transport ;}
A0196	deletion	{Hs10092627; KOG0268 Sof1-like rRNA processing protein (contains WD40 repeats） A RNA processing and modification ;}

A0198	deletion	{SPBC4C3.05c; KOG0262 RNA polymerase I, large subunit K Transcription ;}
A0203	deletion	{Hs4507791; KOG0420 Ubiquitin-protein ligase O Posttranslational modification, protein turnover, chaperones ;}
A0204	deletion	{Hs22060712; KOG3467 Histone H4 B Chromatin structure and dynamics ;}
A0205	deletion	{Hs17442169; KOG1745 Histones H3 and H4 B Chromatin structure and dynamics ;}
A0206	deletion	{7298219; KOG2317 Putative translation initiation inhibitor UK114/IBM1 J Translation, ribosomal structure and biogenesis ;}
A0208	deletion	{SPAC644.14c; KOG1433 DNA repair protein RAD51/RHP55 L Replication, recombination and repair ;}
A0213	deletion	{YIL074c; KOG0068 D-3-phosphoglycerate dehydrogenase, D-isomer-specific 2-hydroxy acid dehydrogenase superfamily E Amino acid transport and metabolism ;}
A0218	deletion	{SPAC3G6.06c; KOG2519 5'-3' exonuclease L Replication, recombination and repair ;}
A0241	duplication	{Hs17999539; KOG0924 mRNA splicing factor ATP-dependent RNA helicase A RNA processing and modification ;}
A0244	duplication	{Hs6912520; KOG2457 A/G-specific adenine DNA glycosylase L Replication, recombination and repair ;}
A0253	duplication	{Hs13653804; KOG3353 60S ribosomal protein L22 J Translation, ribosomal structure and biogenesis ;}
A0255	duplication	{Hs11321607; KOG0744 AAA+-type ATPase O Posttranslational modification, protein turnover, chaperones ;}
A0257	duplication	{SPAC869.05c; KOG0236 Sulfate/bicarbonate/oxalate exchanger SAT-1 and related transporters (SLC26 family） P Inorganic ion transport and metabolism ;}
A0260	duplication	{YFR032c-a; KOG3504 60S ribosomal protein L29 J Translation, ribosomal structure and biogenesis ;}
A0261	duplication	{YDR298c; KOG1662 Mitochondrial F1F0-ATP synthase, subunit OSCP/ATP5 C Energy production and conversion ;}
A0266	duplication	{SPAPB2B4.02; KOG0911 Glutaredoxin-related protein O Posttranslational modification, protein turnover, chaperones ;}
A0268	duplication	{SPAC6F12.16c; KOG0948 Nuclear exosomal RNA helicase MTR4, DEAD-box superfamily A RNA processing and modification ;}
A0273	duplication	{SPBC13E7.01; KOG2140 Uncharacterized conserved protein R General function prediction only ;}
A0274	duplication	{SPBC16D10.09; KOG1636 DNA polymerase delta processivity factor (proliferating cell nuclear antigen） L Replication, recombination and repair ;}
A0276	duplication	{SPBC31F10.06c; KOG0077 Vesicle coat complex COPII, GTPase subunit SAR1 U Intracellular trafficking, secretion, and vesicular transport ;}
A0279	duplication	{SPBC1773.17c; KOG0069 Glyoxylate/hydroxypyruvate reductase (D-isomer-specific 2-hydroxy acid dehydrogenase superfamily） C Energy production and conversion ;}
A0286	duplication	{SPBC119.14; KOG4141 DNA repair and recombination protein

	RAD52/RAD22 L Replication, recombination and repair ;}
A0287 duplication	{YDR331w; KOG1349 Gpi-anchor transamidase O Posttranslational modification, protein turnover, chaperones ;}
A0291 duplication	{YMR002w; KOG4090 Uncharacterized conserved protein S Function unknown ;}
A0296 duplication	{SPAC323.02c; KOG0176 20S proteasome, regulatory subunit alpha type PSMA5/PUP2 O Posttranslational modification, protein turnover, chaperones ;}
A1643 deletion	{SPBPJ4664.04; KOG0292 Vesicle coat complex COPI, alpha subunit U Intracellular trafficking, secretion, and vesicular transport ;}
A2655 deletion	{SPAC1687.03c; KOG0991 Replication factor C, subunit RFC2 L Replication, recombination and repair ;}
A2661 deletion	{SPCC24E4.01; KOG3076 5'-phosphoribosylglycinamide formyltransferase G Carbohydrate transport and metabolism ;}
A2665 deletion	{SPAC637.05c; KOG1351 Vacuolar H^+-ATPase V1 sector, subunit B C Energy production and conversion ;}
A2668 deletion	{Hs4506003; KOG0374 Serine/threonine specific protein phosphatase PP1, catalytic subunit TR Signal transduction mechanisms ; General function prediction only ;}
A2672 deletion	{7297689; KOG0004 Ubiquitin/40S ribosomal protein S27a fusion J Translation, ribosomal structure and biogenesis ;}
A2684 deletion	{CE02385; KOG1773 Stress responsive protein R General function prediction only ;}
A2685 deletion	{At1g20630; KOG0047 Catalase P Inorganic ion transport and metabolism ;}
A2687 deletion	{YOR234c; KOG0887 60S ribosomal protein L35A/L37 J Translation, ribosomal structure and biogenesis ;}
A2688 deletion	{SPCP1E11.08; KOG3163 Uncharacterized conserved protein related to ribosomal protein S8E R General function prediction only ;}
A2691 deletion	{SPCC13B11.01; KOG0023 Alcohol dehydrogenase, class V Q Secondary metabolites biosynthesis, transport and catabolism ;}
A2692 deletion	{SPCC330.09; KOG2321 WD40 repeat protein R General function prediction only ;}
A3960 deletion	{SPCC13B11.01; KOG0023 Alcohol dehydrogenase, class V Q Secondary metabolites biosynthesis, transport and catabolism ;}
A0821 deletion	{SPBC354.12; KOG0657 Glyceraldehyde 3-phosphate dehydrogenase G Carbohydrate transport and metabolism ;}
A0845 deletion	A0846
A1505 deletion	{YJR096w; KOG1577 Aldo/keto reductase family proteins R General function prediction only ;}
A1817 deletion	{SPAC9.09; KOG2263 Methionine synthase II (cobalamin-independent） E Amino acid transport and metabolism ;}
A2501 deletion	{YNL241c; KOG0563 Glucose-6-phosphate 1-dehydrogenase G Carbohydrate transport and metabolism ;}
A3219 deletion	{Hs14727498; KOG0882 Cyclophilin-related peptidyl-prolyl cis-trans

A3866 deletion	isomerase O Posttranslational modification, protein turnover, chaperones ;} {YGL167c; KOG0202 Ca^{2+} transporting ATPase P Inorganic ion transport and metabolism ;}

Table S7 The common differentially expressed genes of the 4 groups（annotable）

name	remark
TRINITY_DN1924_c0_g1	Deoxyhypusine synthase
TRINITY_DN1614_c0_g1	Argininosuccinate lyase
TRINITY_DN4317_c0_g1	Glutathione S-transferase [EC:2.5.1.18]
TRINITY_DN3077_c0_g1	MFS transporter
TRINITY_DN1792_c0_g1	Nitrogen compound metabolic process
TRINITY_DN3287_c0_g1	Extracellular polysaccharide metabolic process
TRINITY_DN2147_c0_g1	Misacylated tRNA(Ala) deacylase
TRINITY_DN1825_c0_g1	aspartic-type endopeptidase activity
TRINITY_DN1172_c0_g1	Uncharacterized protein
TRINITY_DN3290_c0_g1	Cellular response to oxidative stress;
TRINITY_DN1839_c0_g1	Acid phosphatase
TRINITY_DN3430_c0_g1	delta8-fatty-acid desaturase
TRINITY_DN3832_c0_g1	Paladin [synthetic construct]
TRINITY_DN4210_c0_g2	Large subunit ribosomal protein L34e
TRINITY_DN1668_c0_g3	Egulation of cyclin-dependent protein serine/threonine kinase activity
TRINITY_DN2348_c0_g2	Cell division control protein 7
TRINITY_DN2752_c0_g2	MFS transporter, FHS family, L-fucose permease
TRINITY_DN2727_c0_g1	Ferritin-like domain

2 Supplementary Figure

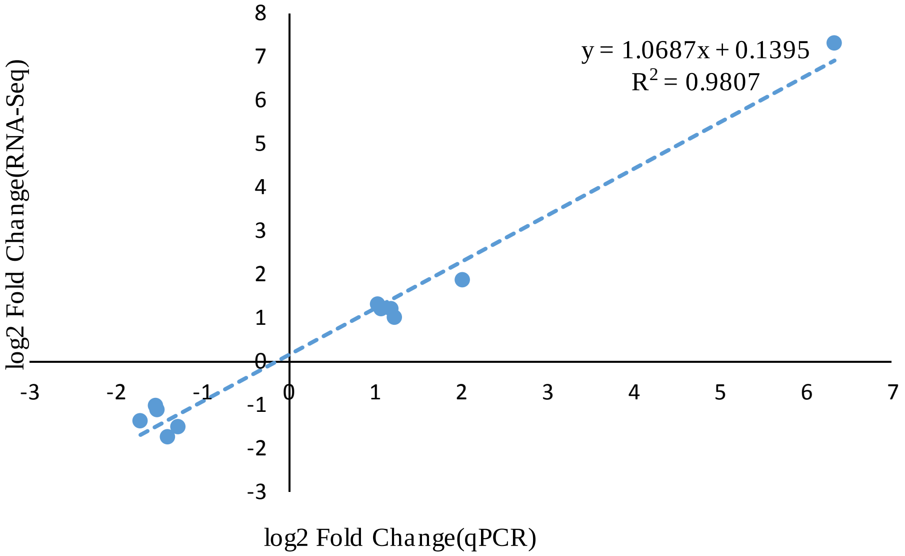

Supplementary Figure 1 qRT-PCR verification RNA-Seq result analysis chart

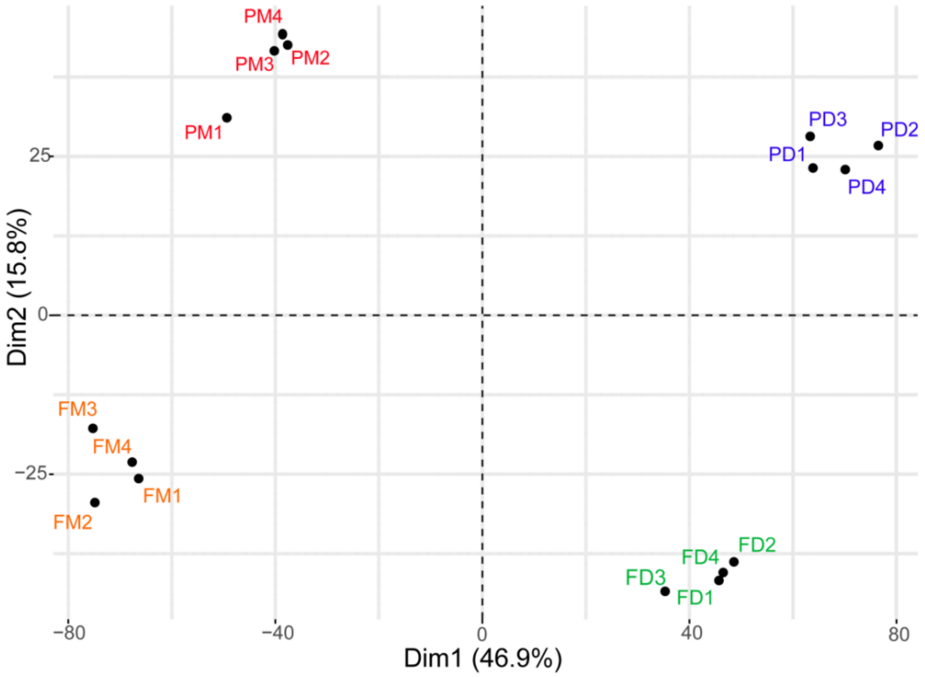

Supplementary Figure 2 The analysis of parallelism in RNA-seq.

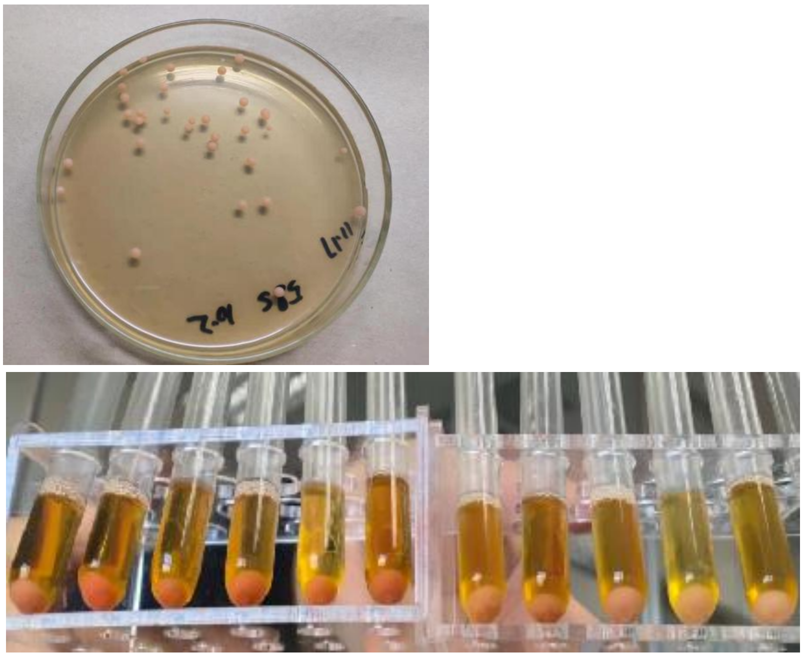

Supplementary Figure 3 The phenotypic of mutants in agar plate and tube of YPD.

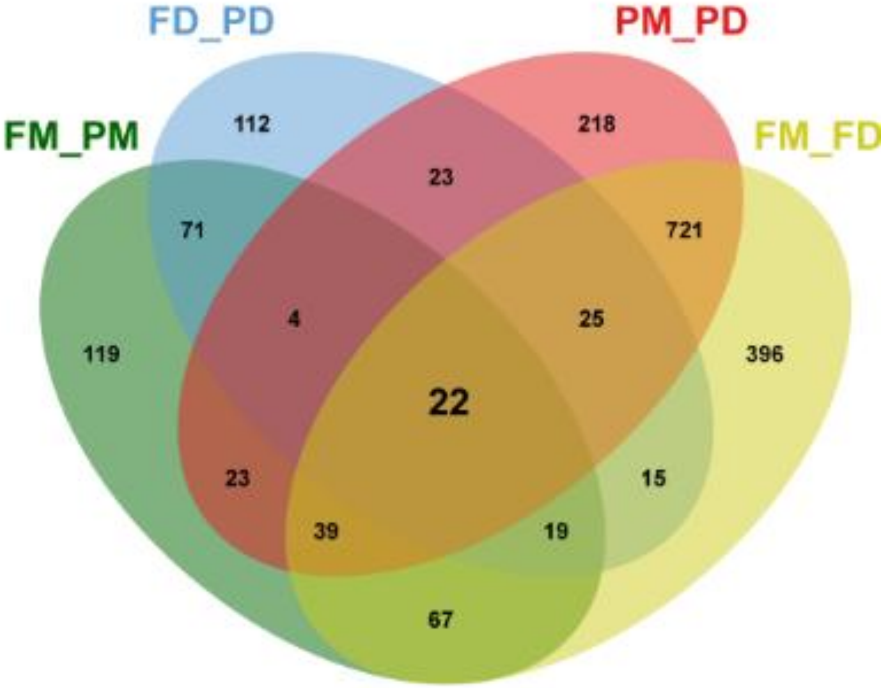

Supplementary Figure 4 Venn plot of 4 groups of differentially expressed genes.
